# Clonality Analysis of *Helicobacter pylori* in Patients Isolated from Several Biopsy Specimens and Gastric Juice in a Japanese Urban Population by Random Amplified Polymorphic DNA Fingerprinting

**DOI:** 10.1155/2013/721306

**Published:** 2013-11-17

**Authors:** Nariaki Toita, Shin-ichi Yokota, Nobuhiro Fujii, Mutsuko Konno

**Affiliations:** ^1^Department of Pediatrics, Sapporo Kosei General Hospital, North-3, East-8, Chuo-ku, Sapporo 060-0033, Japan; ^2^Department of Microbiology, Sapporo Medical University School of Medicine, South-1, West-17, Chuo-ku, Sapporo 060-8556, Japan

## Abstract

*Background*. The number of *Helicobacter pylori* clones infecting a single host has been discussed in numerous reports. The number has been suggested to vary depending on the regions in the world. *Aim*. The purpose of this study was to examine the number of clones infecting a single host in a Japanese urban population. *Materials and Methods*. Thirty-one Japanese patients undergoing upper gastrointestinal endoscopy were enrolled in this study. *H. pylori* isolates (total 104 strains) were obtained from biopsy specimens (antrum, corpus, and duodenum) and gastric juice. Clonal diversity was examined by the random amplified polymorphic DNA (RAPD) fingerprinting method. *Results*. The RAPD fingerprinting patterns of isolates from each patient were identical or very similar. And the isolates obtained from several patients with 5- to 9-year intervals showed identical or very similar RAPD patterns. *Conclusion*. Each Japanese individual of an urban population is predominantly infected with a single *H. pylori* clone.

## 1. Introduction

*Helicobacter pylori* is a bacterial pathogen responsible for the development of numerous gastrointestinal disorders, including gastritis, gastric and duodenal ulcers, gastric adenocarcinoma, and gastric lymphoma [1–3]. Although eradication of *H. pylori* may prevent those complications later in life, failure of antibiotic treatment is often caused by antibiotic-resistant *H. pylori* strains. The prevalence of resistance to antibiotics appears to be increasing, so susceptibility testing for antibiotics plays an important role in treatment [4, 5]. If plural clones of *H. pylori*, including antibiotic-resistant clones, coexist in a single patient, failure of eradication may occur due to microbial substitution despite positive results of an antibiotic susceptibility test. It seems that the number of *H. pylori* clones isolated from a single host varies depending on the geographic region [6–13]. Several genotyping methods have been applied to *H. pylori* for epidemiological analysis. Among them, a convenient procedure, random amplified polymorphic DNA (RAPD) fingerprinting method, has been used to analyze *H. pylori* isolated from the stomach in an attempt to ascertain whether or not multiple clones are present in a single host [8–13].

Early studies showed that a single primer could be used to distinguish *H. pylori* clones by RAPD fingerprinting profiles [9–11]. However, recent studies have used a combination of several primers to more precisely discriminate unrelated clones [13–15].

The purpose of this study was to investigate *H. pylori* clone diversity in Japanese patients by sampling from multiple sites of the stomach and gastric juice derived from a single patient, using the RAPD fingerprinting method with several primers.

## 2. Materials and Methods

### 2.1. Subjects

Thirty-one donors (14 males and 17 females) who came to Sapporo Kosei General Hospital (Sapporo, Japan) for upper gastrointestinal endoscopy were enrolled in this study as shown in Tables 1, 2, and 3. The subjects were all Japanese. *H. pylori* infection was diagnosed by the stool antigen test (Premier Platinum HpSA PLUS, Meridian Bioscience, Cincinnati, OH, USA). The patients received neither antibiotics, proton pump inhibitors, nor nonsteroidal anti-inflammatory drugs within 1 month before the specimens were taken. Biopsy specimens were taken (from antrum, corpus, and duodenum) using a sterilized endoscope. The biopsy forceps were disinfected by immersion in 0.05% phtharal for 5 min and then rinsed with water for each specimen collection. And gastric juice was also obtained. Informed consent was obtained from all patients. This work was approved by the Review Board of Sapporo Kosei General Hospital.

### 2.2. Culture of Biopsy Samples

The isolation and identification of *H. pylori* from biopsy specimens and gastric juice were described elsewhere [14, 15]. *H. pylori* isolates were cultured on *Helicobacter*-selection agar plates (Nissui Pharmaceutical, Tokyo, Japan) at 37°C in a microaerophilic atmosphere (10% O_2_ and 15% CO_2_).

### 2.3. RAPD Fingerprinting Method

Extraction of *H. pylori* genomic DNA from bacterial cells and PCR-based RAPD analysis were performed in accordance with the processes described previously [15]. PCR was carried out using 20 ng template DNA, 20 pmol primer, and HotStarTaq master mix (Qiagen, Hilden, Germany). The PCR primers were selected from random primers of DNA Oligomer set A-4 (NIPPON GENE, Tokyo, Japan). Out of the 12 primers (A01 to A12), four primers, A04 (5′-ATCAGCGCACCA-3′), A07 (5′-TGCCTCGCACCA-3′), A08 (5′-GCCCCGTTAGCA-3′), and A11 (5′-GATGGATTTGGG-3′) were suitable for this study. A GeneAmp PCR system 9600-R cycler (Applied Biosystems, Grand Island, NY, USA) was used for amplification. The cycling program was 35 cycles of 94°C for 2 min, 38°C for 2 min, and 72°C for 2 min, followed by a final incubation at 72°C for 10 min. The products were analyzed by 2% agarose gel electrophoresis. The EZ Load 100 bp ladder marker (Bio-Rad, Hercules, CA, USA) or the pHY marker (Takara, Shiga, Japan) was used as a size marker.

## 3. Results

**Figure 1:**
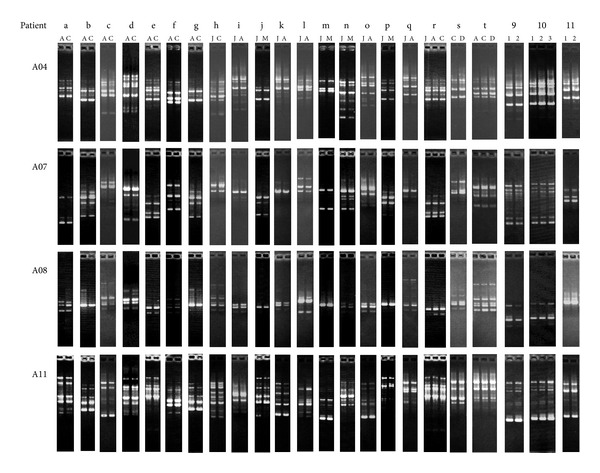
RAPD fingerprinting patterns of *H. pylori *isolates from patients (a to t in Table 1 and 9 to 11 in Table 2) obtained by RAPD analysis with primers A04, A07, A08, and A11. A: antrum, C: corpus, D: duodenum, J: gastric juice, and M: mucosa.

Endoscopic examination and histopathological examination of the biopsy specimens were performed for diagnosis. For twenty patients, an *H. pylori *colony was isolated and cultured from each biopsy specimen (from the antrum, corpus, and duodenum) and from gastric juice (Table 1). All isolates were subjected to RAPD fingerprinting by using 4 primers (A04, A07, A08 and A11). Although microheterogeneity was observed, RAPD profiles obtained by four primers showed identical or very similar patterns among all specimens derived from a single patient (Figure 1).

 For three patients, *H. pylori* was isolated and cultured from biopsy specimens and/or gastric juice that were obtained with long time intervals (5 to 9 years) (Table 2). These isolates derived from each patient showed identical or very similar RAPD patterns (Figure 1).

**Figure 2:**
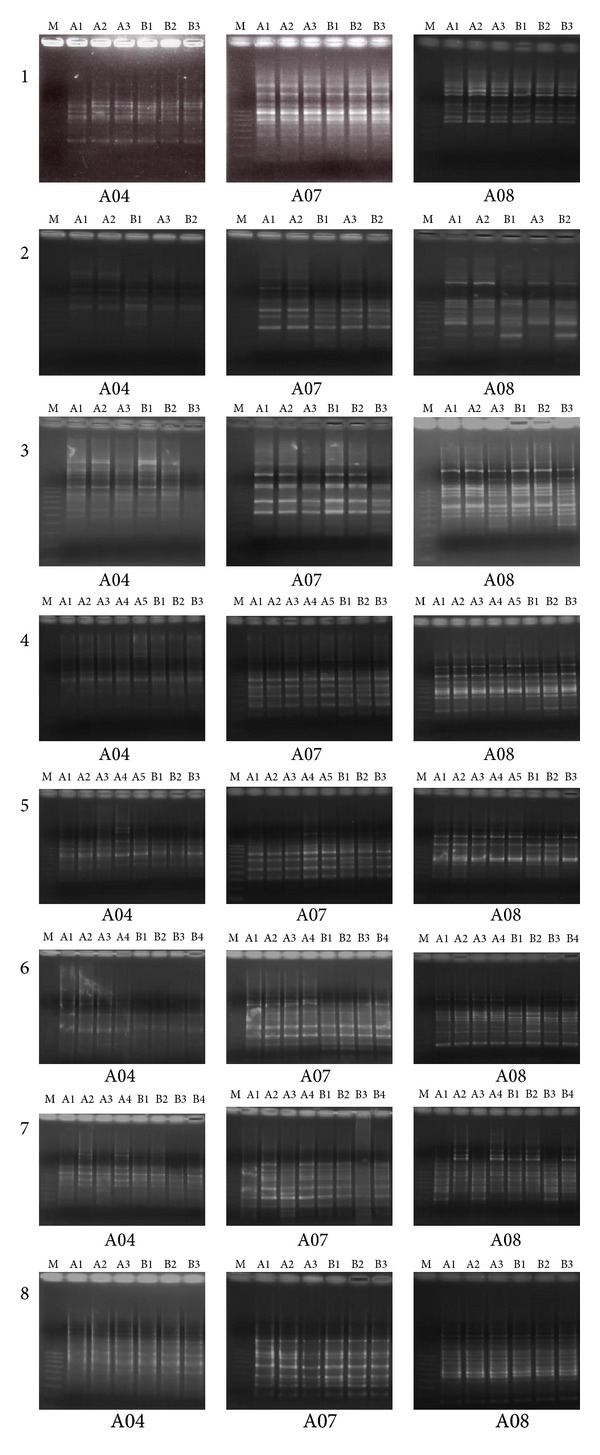
RAPD fingerprint patterns of genomic DNAs from *H. pylori *isolates from eight patients (No. 1–8) obtained by RAPD analysis with primers A04, A07, A08, and A11. DNA samples were obtained from the antrum (A series) and corpus (B series). No. 4 and 5 are members of the same family. Data for primer A11 are not shown. A 100 bp DNA ladder was used as a size marker (lane M).

 For eight patients, several (2 to 5) colonies were isolated from each specimen (antrum and corpus) (Table 3). RAPD fingerprinting patterns with four primers (A04, A07, A08, and A11) were also identical or very similar in isolates derived from a single patient (Figure 2; and the results for A11 are not shown).

 The results strongly suggested that all of the patients were infected with one dominant clone in the stomach. *H. pylori* isolates obtained from two patients belonging to the same family showed identical fingerprints, indicating infection with the same strain of *H. pylori* (No. 4 and 5) (Figure 2). The results were not caused by contamination because the endoscopy and biopsy for each subject were carried out on separate days.

## 4. Discussion

A number of studies on the predominance of *H. pylori* clones have been carried out [6–13, 16]. In the present study, RAPD fingerprinting patterns of the isolates from biopsy specimens (from the antrum, corpus, and duodenum) and gastric juice of a single Japanese patient were identical or very similar, strongly suggesting that each patient was colonized by a single *H. pylori* clone. There are diverse reports on the clonality of *H. pylori *infection. Some reports describe that colonization with *H. pylori *multiple clones seems to be common [6, 10–12]. Other reports describe that colonization with plural clones appears to be relatively rare [7–9, 13]. Prevalence of *H. pylori* infection varies depending on the country, age, and socioeconomic and hygienic status [17, 18]. Clonality of *H. pylori* may also be related to such factors. Interestingly, Hua et al. reported that 58 patients in Singapore harbored a single *H. pylori* clone [9], whereas Norazah et al. reported that 31.3% of individuals had been found to be colonized with multiple clones in Malaysia, which is located in the same southern part of Malay Peninsula [12]. It is thought that infection from the environment is rare in urban area and that intrafamilial infection is the major route. Previously, we investigated the infection route of *H. pylori* in Japanese children by RAPD analysis [14]. Results of that study suggested that 76% of the Japanese children acquired *H. pylori* through intrafamilial infections and that about 90% of the intrafamilial infections were mother-to-child infections. This might cause clonal infection in a single host. *H. pylori* prevalence has reached 70% or more in developing countries, such as 71.7% in China, 92% in Bangladesh, 80% in Kazakhstan and 80% in India, while that in Japan has decreased to 39.3% [19–23]. Moreover, *H. pylori* infection in the Japanese less than 50 years of age is low prevalence. It may be one cause of the single clone infection (as shown in this study), because of few opportunities of infection, as well as other developed countries. And mother-to-child infection is predominant in Japan as indicated by our studies [14, 15]. In addition, the patients examined in this study live in Sapporo city, which is the fourth largest city in Japan, and its neighbors. So the socioeconomic and hygiene status are considered relatively high. Furthermore, strains that were considered to be originated from the same clone were isolated from the same patients with long time intervals, 5 to 9 years (Table 2). This suggests that a single clone persistently colonizes an individual who has been infected with *H. pylori*.

Among several genotyping methods applied to *H. pylori*, RAPD-PCR is considered to be useful because it is a simple, rapid, and low-cost method for distinguishing one *H. pylori* clone from another [15]. Microheterogeneity of RAPD fingerprinting patterns may occur, as found in our study, and this is thought to originate from minor alternations that have occurred in the genome of a *H. pylori *clone. To obtain accurate results, we use three to four PCR primers for RAPD experiments. In recent studies, Roma-Giannikou et al. used two primers for 32 subjects [24], and Dubois et al. used four primers to precisely distinguish between isolates [25]. Thus, RAPD fingerprinting analysis with careful attention is one of the best techniques for determining *H. pylori *clones.

## 5. Conclusions

We indicated that RAPD fingerprinting patterns of *H. pylori* isolated from biopsy specimens (from the corpus, antrum, and duodenum) and gastric juice of Japanese individuals in an urban population were identical or very similar. These results strongly suggest that Japanese individuals are predominantly infected with a single *H. pylori *clone.

## Figures and Tables

**Table 1:** Characteristics of patients from whom plural isolates, which were thought to be originated from the same clone, were obtained from different specimens.

Isolates derived from	Number of patients	Patients (gender, years of age, and disease)*
Antrum and corpus	7	F 14, CG (a); F 44, CG (b); F 46, CG (c); F 46, CG (d); M 11 CG (e); M 12, CG (f); M 13, CG (g)
Gastric juice and mucosa	10	F 11, CG (h); F 18, CG (i); F 38, CG (j); F 39, CG (k); F 42, GU (l); F 44, CG (m); F 47, GU (n); F 54, CG (o); M 8Y, DU (p); M 14, DU (q)
Antrum, corpus, and gastric juice	1	F 34, GU (r)
Corpus and duodenum	1	M 48, CG (s)
Antrum, corpus, and duodenum	1	M 28, DU (t)

*M: male, F: female, CG: chronic gastritis, GU: gastric ulcer, and DU: duodenal ulcer.

( ): RAPD fingerprinting patterns of these patients that are shown in Figure 1.

**Table 2:** Characteristics of patients from whom plural isolates, which were thought to be originated from the same clone, were obtained from specimens obtained with long intervals.

Patient	Age (years)	Gender	Disease	Strains (source and time of isolation)
9	8	M	CG	(1)* gastric juice (2005. 10)
	13			(2) antrum (2010. 6)
10	46	F	CG	(1) gastric juice (2003. 5)
	54			(2) gastric juice (2012. 7)
	55			(3) antrum (2012. 11)
11	17	M	CG	(1) gastric juice (2003. 8)
	26			(2) antrum (2012. 11)

M: male, F: female, and CG: chronic gastritis.

Patients 10 and 11 are parent and child. These strains are considered to be originated from the same clone.

*RAPD fingerprinting patterns of these strains are shown in Figure 1. The numbers correspond to lane numbers of each patient.

**Table 3:** Characteristics of patients from whom plural isolates were obtained from a biopsy specimen.

Patient	Age (year)	Gender	Disease	Number of isolates	
				Antrum	Corpus
1	9	M	CG	3	3
2	55	M	CG	3	2
3	35	M	GU	3	3
4	43	M	GU	5	3
5	32	F	CG	5	3
6	49	F	CG	4	4
7	10	M	CG	4	4
8	35	F	CG	3	3

M: male, F: female, CG: chronic gastritis, and GU: gastric ulcer.

## List of Abbreviations

PCR: Polymerase chain reaction
PAPD: Randomly amplified polymorphic DNA.

## References

[1] Uemura N, Okamoto S, Yamamoto S (2001). *Helicobacter pylori* infection and the development of gastric cancer. *The New England Journal of Medicine*.

[2] Suerbaum S, Michetti P (2002). *Helicobacter pylori* infection. *The New England Journal of Medicine*.

[3] Graham DY (1997). *Helicobacter pylori* infection in the pathogenesis of duodenal ulcer and gastric cancer: a model. *Gastroenterology*.

[4] Graham DY (1998). Antibiotic resistance in *Helicobacter pylori*: implications for therapy. *Gastroenterology*.

[5] Jenks PJ, Edwards DI (2002). Metronidazole resistance in *Helicobacter pylori*. *International Journal of Antimicrobial Agents*.

[6] Kim YS, Kim N, Kim JM (2009). *Helicobacter pylori* genotyping findings from multiple cultured isolates and mucosal biopsy specimens: strain diversities of *Helicobacter pylori* isolates in individual hosts. *European Journal of Gastroenterology and Hepatology*.

[7] Miehlke S, Thomas R, Guiterrez O, Graham DY, Go MF (1999). DNA fingerprinting of single colonies of *Helicobacter pylori* from gastric cancer patients suggests infection with a single predominant strain. *Journal of Clinical Microbiology*.

[8] Hirschl AM, Richter M, Makristathis A (1994). Single and multiple strain colonization in patients with *Helicobacter pylori*-associated gastritis: detection by macrorestriction DNA analysis. *Journal of Infectious Diseases*.

[9] Hua J, Ng MC, Yeoh KG, Ho B (1999). Predominance of a single strain of *Helicobacter pylori* in gastric antrum. *Helicobacter*.

[10] Kim JJ, Kim JG, Kwon DH (2003). Mixed-infection of antibiotic susceptible and resistant *Helicobacter pylori* isolates in a single patient and underestimation of antimicrobial susceptibility testing. *Helicobacter*.

[11] Jorgensen M, Daskalopoulos G, Warburton V, Mitchell HM, Hazell SL (1996). Multiple strain colonization and metronidazole resistance in *Helicobacter pylori*-infected patients: identification from sequential and multiple biopsy specimens. *Journal of Infectious Diseases*.

[12] Norazah A, Wan Rasinah Z, Zaili Z, Aminuddin A, Ramelah M (2009). Analysis of PCR-RAPD DNA and antibiotic susceptibility profiles of antrum and corpus isolates of *Helicobacter pylori* from Malaysian patients. *Malaysian Journal of Pathology*.

[13] Lee YC, Lee S-Y, Pyo JH, Kwon DH, Rhee JC, Kim JJ (2005). Isogenic variation of *Helicobacter pylori* strain resulting in heteroresistant antibacterial phenotypes in a single host in vivo. *Helicobacter*.

[14] Konno M, Yokota S, Suga T, Takahashi M, Sato K, Fujii N (2008). Predominance of mother-to-child transmission of *Helicobacter pylori* infection detected by random amplified polymorphic DNA fingerprinting analysis in Japanese families. *Pediatric Infectious Disease Journal*.

[15] Konno M, Fujii N, Yokota S (2005). Five-year follow-up study of mother-to-child transmission of *Helicobacter pylori* infection detected by a random amplified polymorphic DNA fingerprinting method. *Journal of Clinical Microbiology*.

[16] Marshall DG, Chua A, Keeling PWN, Sullivan DJ, Coleman DC, Smyth CJ (1995). Molecular analysis of *Helicobacter pylori* populations in antral biopsies from individual patients using randomly amplified polymorphic DNA (RAPD) fingerprinting. *FEMS Immunology and Medical Microbiology*.

[17] Bardhan PK (1997). Epidemiological features of *Helicobacter pylori* infection in developing countries. *Clinical Infectious Diseases*.

[18] Farinha P, Gascoyne RD (2005). *Helicobacter pylori* and MALT lymphoma. *Gastroenterology*.

[19] Fujisawa T, Kumagai T, Akamatsu T, Kiyosawa K, Matsunaga Y (1999). Changes in seroepidemiological pattern of *Helicobacter pylori* and hepatitis A virus over the last 20 years in Japan. *American Journal of Gastroenterology*.

[20] Li X, Zou D, Ma X (2010). Epidemiology of peptic ulcer disease: endoscopic results of the systematic investigation of gastrointestinal disease in China. *American Journal of Gastroenterology*.

[21] Ahmad MM, Rahman M, Rumi AK (1997). Prevalence of *Helicobacter pylori* in asymptomatic population—a pilot serological study in Bangladesh. *Journal of Epidemiology*.

[22] Nurgalieva ZZ, Malaty HM, Graham DY (2002). *Helicobacter pylori* infection in Kazakhstan: effect of water source and household hygiene. *American Journal of Tropical Medicine and Hygiene*.

[23] Ahmed KS, Khan AA, Ahmed I (2007). Impact of household hygiene and water source on the prevalence and transmission of *Helicobacter pylori*: a South Indian perspective. *Singapore Medical Journal*.

[24] Roma-Giannikou E, Karameris A, Balatsos B (2003). Intrafamilial spread of *Hhelicobacter pylori*: a genetic analysis. *Helicobacter*.

[25] Dubois A, Berg DE, Incecik ET (1999). Host specificity of *Helicobacter pylori* strains and host responses in experimentally challenged nonhuman primates. *Gastroenterology*.

